# The Importance of Vitamin K and the Combination of Vitamins K and D for Calcium Metabolism and Bone Health: A Review

**DOI:** 10.3390/nu16152420

**Published:** 2024-07-25

**Authors:** Jan O. Aaseth, Trine Elisabeth Finnes, Merete Askim, Jan Alexander

**Affiliations:** 1 Department of Research, Innlandet Hospital Trust, P.O. Box 104, N-2381 Brumunddal, Norway; 2 Faculty of Health and Social Sciences, Inland Norway University of Applied Sciences, N-2418 Elverum, Norway; 3 Department of Medicine, Innlandet Hospital Hamar, P.O. Box 4453, N-2326 Hamar, Norway; trine.e.finnes@sykehuset-innlandet.no; 4 Department of Endocrinology, Oslo University Hospital, P.O. Box 4950 Nydalen, N-0424 Oslo, Norway; 5 Independent Researcher, Bromstadvegen 43, N-7045 Trondheim, Norway; merete.askim@gmail.com; 6 Norwegian Institute of Public Health, P.O. Box 222 Skøyen, N-0213 Oslo, Norway; jan.alexander@fhi.no

**Keywords:** vitamin K, osteocalcin, matrix Gla protein, osteoporosis, vascular calcification, bone loss

## Abstract

The aim of the present review is to discuss the roles of vitamin K (phylloquinone or menaquinones) and vitamin K-dependent proteins, and the combined action of the vitamins K and D, for the maintenance of bone health. The most relevant vitamin K-dependent proteins in this respect are osteocalcin and matrix Gla-protein (MGP). When carboxylated, these proteins appear to have the ability to chelate and import calcium from the blood to the bone, thereby reducing the risk of osteoporosis. Carboxylated osteocalcin appears to contribute directly to bone quality and strength. An adequate vitamin K status is required for the carboxylation of MGP and osteocalcin. In addition, vitamin K acts on bone metabolism by other mechanisms, such as menaquinone 4 acting as a ligand for the nuclear steroid and xenobiotic receptor (SXR). In this narrative review, we examine the evidence for increased bone mineralization through the dietary adequacy of vitamin K. Summarizing the evidence for a synergistic effect of vitamin K and vitamin D3, we find that an adequate supply of vitamin K, on top of an optimal vitamin D status, seems to add to the benefit of maintaining bone health. More research related to synergism and the possible mechanisms of vitamins D3 and K interaction in bone health is needed.

## 1. Introduction

Increased fracture risk characterizes the skeletal disorder osteoporosis [1]. The WHO criteria state that osteoporosis exists in cases with 2.5 or more standard deviations of lower bone mineral density (BMD) than the mean peak BMD for healthy young adults [2]. Primary osteoporosis is induced by estrogen deficiency in postmenopausal women, and it is seen in men as well as in women in older age groups [3]. Secondary osteoporosis is a result of a particular pathology or of treatment with some pharmacological agents [4], for instance in rheumatic diseases and after bariatric surgery or in corticosteroid medication [5,6,7]. Recent meta-analyses have indicated the worldwide prevalence of osteoporosis of almost 20%; its prevalence, however, is characterized by significant geographic variations [8,9]. In Europe, osteoporotic fractures are considered one of the most significant pathological conditions responsible for reduced adjusted life years [10], and the burden of osteoporosis is anticipated to increase further in the coming decades [11]. 

Various exogenous factors including smoking, alcohol consumption, dietary habits, and body weight will modify the risk of the disease [12]. Dietary factors include the intake of calcium and of the vitamins D and K [13]. The role of nutrient deficiencies in osteoporosis has also been confirmed by the observations of postoperative osteoporosis in subjects treated with bariatric surgery [6,7]. It is well known that calcium (Ca) and phosphorus (P) represent the key minerals composing the inorganic bone matrix, viz., the hydroxyapatite (Ca_10_(PO_4_)_6_(OH)_2_) [14]. The metabolism of these minerals is strictly regulated by the parathyroid hormone (PTH) and 1,25-dihydroxyvitamin D (1,25(OH)_2_D), which operate in collaboration with vitamin K [6,15]. The roles of Ca and vitamin D as key nutrients have been widely discussed in previous excellent reviews [16]. Interestingly, although Ca is essential for bone health, systematic analysis shows that populations from developing countries with low Ca intake (below about 900 mg/day) are characterized by lower risk of osteoporotic bone fractures as compared to developed countries [17]. Furthermore, it has been observed that the association between increased Ca intake and BMD appears clinically irrelevant, with a baseline intake of at least 900 mg/day [18]. Apparently, Ca homeostasis, rather than the dietary amount of Ca, represents a key factor linking Ca to osteoporosis under these circumstances. By being regulators of Ca homeostasis, vitamins D and K appear to be essential agents in the preventive treatment of bone loss [19,20]. However, the mechanisms of the multiple effects of these vitamins on bone physiology are still insufficiently characterized, and epidemiological studies sometimes reveal contradictory results. 

The objective of the present study is to review recent epidemiological and clinical data on the association of vitamin K deficiency and combined vitamin K and D deficiencies with osteoporosis and to then discuss the mechanisms underlying these associations. 

The present narrative review is based on a search of PubMed, Medline, and Google Scholar, in addition to our own research. The keywords used in the searches were Vitamin K, Vitamin D, AND Osteoporosis or Bone mineral density (BMD). The search was limited to papers in the English language published in the period 2000–2023.

## 2. Vitamin K as Activator of Osteocalcin and the Bone Health

Vitamin K is a lipid-soluble vitamin that is found in various forms, viz., vitamins K1 (phylloquinone) and K2 (menaquinones, MK). The derivative without the side chain is called menadione or K3 and is, as such, without vitamin K activity [21]. It has been suggested that K1, the main form in the liver, is of particular importance for the synthesis and carboxylation of several vitamin K-dependent coagulation factors, while K2 appears to play a more important role in peripheral tissues with a beneficial effect on skeletal diseases [22].

**Figure 1:**
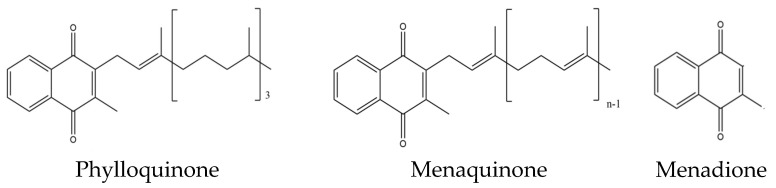
The structure of vitamin K1 (phylloquinone) and K2 (menaquinone, MK), where the number n indicates the number of isoprenyl groups (4–10) at the C3 position. The structure without a side chain is called menadione (vitamin K3) and is, as such, without vitamin K activity.

In particular, vitamin K2 (Figure 1) has been found to be involved in bone remodeling and bone health [23,24]. Most commonly, vitamin K2 exists in the form of menaquinone-4 to -10 (often denoted as MK-4 to MK-10, respectively) [25], where the numbers (n in Figure 1) indicate the number of isoprenyl groups at the C3 position [26].

Vitamin K is a cofactor for an endoplasmic reticulum carboxylase that operates by activating some glutamic-acid-containing proteins (glu-proteins) into gamma-carboxyglutamic-acid-containing proteins (gla-proteins). The gla-amino acid is excreted in the urine when the protein is catabolized. 

**Figure 2:**
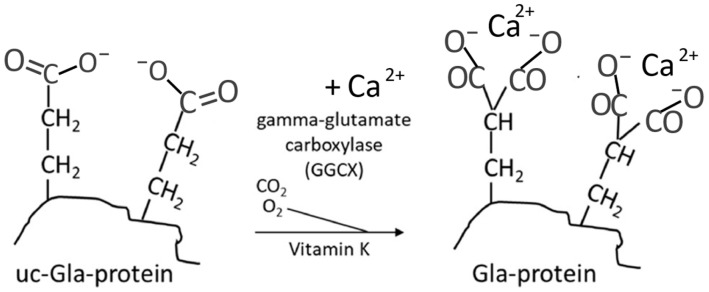
Carboxylation of the glutamate unit of a protein, e.g., of osteocalcin, making it able to bind calcium. (Figure adapted from Aaseth et al., 2023 [26]).

Among these proteins, *osteocalcin* is of particular importance for bone health. Osteocalcin is produced exclusively by osteoblasts. The posttranslational activation involves an enzymatic carboxylation (Figure 2) of three of its glutamic acid units, thereby converting these units into calcium-chelating groups [27]. One of the main functions of the activated osteocalcin appears to involve the import of Ca from the blood and other tissues into the bone for incorporation into the hydroxyapatite, thereby influencing bone mineralization and making the skeleton less susceptible to fractures [27]. One hypothesis states that osteocalcin can strengthen the bone through bridges between the bone components, as it can be bound both to hydroxyapatite and to the matrix protein, osteopontin [28]. 

The chemical state of osteocalcin has been used as a biomarker for vitamin K status. A low serum ratio of carboxylated osteocalcin (cOC) to undercarboxylated osteocalcin (ucOC) or an increased serum concentration of ucOC reflects an inadequate functional vitamin K status, which appears to be associated with bone loss and the increased risk of hip fractures [29]. 

## 3. Observational and Interventional and Studies on Vitamin K and Bone

In general, bone density decreases with age, a process that is usually paralleled by increased vascular calcification [26,30]. A parallel phenomenon is a decline in vitamin K status that has been observed to result in a low ratio of carboxylated (cOC) to undercarboxylated osteocalcin (ucOC) [31]. 

### 3.1. Observational Studies

Several studies have reported that low intake of vitamin K and/or low plasma vitamin K concentration are associated with increased risk of fractures, especially in post-menopausal women. The U.S. Nurses’ Health Study followed more than 72,000 women for 10 years and reported that women whose K1 intakes were lower than 109 μg/day had a 30% higher risk for hip fracture compared to women with intakes equal to or above 109 μg/day [32]. Associations between fractures and vitamin K1 levels have also been observed in elderly Norwegians [33] and in elderly Asians [34] of both genders. A similar relationship has been seen in the Japanese general population as observed in a survey of unspecified vitamin K intake and the incidence of hip fractures [35]. Other large observational studies have confirmed these findings [36]. However, other researchers have not found an association of unspecified vitamin K intake and bone fracture risk, e.g., in Hong Kong [37]. Neither has an association been seen between K1 or K2 intake and vertebral fractures in the Hordaland Health Study of men and women in the age group of 70–75 years. However, the intake of K1 in the lowest quartile was associated with an increased risk of hip fractures, while there was no similar association with the intake of K2 [38]. In this Norwegian study, the intake was assessed from food frequency questionnaires, and the estimated intake of K2 was rather low (mean values of below 14 µg/day). Beulens et al. [39] observed an inverse relationship between circulating vitamin K2 (MK-7) levels and the incidence of fractures in Japanese women. In a recent Japanese cohort study, a protective association of dietary vitamin K intake was seen for vertebral fractures in middle-aged and elderly women [40].

### 3.2. Interventional Studies on Vitamin K and Osteoporosis

Most interventional studies have made use of supplementation with vitamin K2. A recent meta-analysis by Ma et al. [41] addressing the impact of vitamin K2 supplementation in postmenopausal women with and without osteoporosis included 16 randomized clinical trials (RCTs). In this meta-analysis, studies that included vitamin K1 were excluded. Primary outcomes were changes in BMD before and after treatment, fracture incidence, and changes in ucOC and cOC. The studies involved supplementation with K2 alone or on top of vitamin D, calcium, or a bisphosphonate. It was concluded that in 10 studies, vitamin K2 supplementation improved lumbar spine BMD; however, in subgroup analysis, only K2 given in combination with other remedies had a significant effect. K2 reduced fracture risk based on the remaining five studies, while one study had been removed due to heterogeneity. K2 also decreased serum ucOC, while there was no change in cOC. 

The effect of vitamin K2 (45 mg MK-4/day for 12 months) in addition to risedronate, investigated in an RCT of 101 elderly women with postmenopausal osteoporosis, showed no significant difference between the risedronate group and the combined therapy group in terms of vertebral fracture incidence [42]. However, in an RCT of postmenopausal women, Hirao et al. [43] found a significant effect of K2 MK-4 (45 mg/day for one year) on lumbar spine BMD, when given on top of alendronate. Møller et al. [44] showed that a six-week period of supplementation with 180 μg/day of vitamin MK-7 in healthy subjects (20–66 years of age) was associated with an increase in serum cOC and a reduction in serum ucOC concentration.

A meta-analysis of 19 RCTs involving 6759 study postmenopausal participants [45], partly overlapping with the meta-analysis of Ma et al. [41], concluded that vitamin K2 played a significant role in the maintenance of vertebral BMD and in the prevention of fractures in the subgroup of postmenopausal women with osteoporosis. But in the non-osteoporotic subgroup, vitamin K2 did not give rise to any significant changes.

In 2022, Zhou et al. [46] reported the results of another meta-analysis of nine RCTs with 6853 study participants, also partly overlapping with the meta-analysis of Ma et al. [41]. Zhou et al. concluded that vitamin K2 plays an important role for the maintenance of BMD. They also summarized the observations showing that the vitamin decreases the levels of ucOC and increases cOC levels upon long-term follow-up. The latter researchers concluded that vitamin K2 supplementation is safe and beneficial in the preventive treatment of osteoporosis for postmenopausal women. 

Apparently, adequate intakes of vitamin K2 improve bone quality in older age groups, which in turn reduces fracture risk, as has been observed in several studies with population groups above the age of 50. However, a beneficial effect of vitamin K supplementation is expected only in individuals with a suboptimal pre-interventional status. This makes interpretations from the present studies difficult, especially interpretation from studies conducted in Japanese populations [42,43], as Japanese populations generally have a sufficient intake of vitamin K2. Of note, variable results have been obtained by intervention, depending upon the study design, whether vitamin K2 was given alone or on top of other treatments such as calcium, vitamin D, or bisphosphonate, and whether treatment groups had osteoporosis or not. Moreover, in some studies, vitamin K2, mostly MK-4, was given in doses of 45 mg/day, which could be considered pharmacological doses as they are far above those necessary from a nutritional point of view. In several studies, vitamin K2 resulted in a reduction in ucOC, indicating a suboptimal or deficient vitamin K status. Although the expression and activation of osteocalcin is apparently related to proper bone function, the precise *mechanisms* behind the impact of vitamin K are still under investigation [47]. 

## 4. Mechanisms of Action of Vitamin K

The important effects of vitamin K on Ca and skeletal homeostasis are known to be mediated through its role as a cofactor for the γ-glutamyl carboxylase enzyme that promotes conversion of glutamate (Glu) residues to gamma-carboxyglutamic (Gla) residues in the post-translational carboxylation of osteocalcin (OC) and matrix Gla protein (MGP) (Figure 2). This may have a significant impact on osteogenesis [48]. Activated MGP with five calcium and three phosphate chelating groups, in cooperation with osteocalcin with three dicarboxylic groups, appears to be essential for an adequate transfer of calcium from the circulation to the bones [28]. In this respect, the action of these vitamin K-dependent proteins (VKDPs) appears to have similarities with the action of the anti-osteoporotic bisphosphonates [49]. However, some additional effects on bone health appear to be dependent on the forms of vitamin K species. For instance, the osteogenic effect of MK-4 is reported to be partly mediated by the activation of the Wnt/β-catenin signaling pathway [50], whereas MK-7 has been shown to increase osteoblast activation through a downregulation of the Runx2 expression [51], indicating that this homologue may also promote osteoblast maturation [52]. 

It has been reported that vitamin K2 as MK-4 and MK-7 is more efficient than vitamin K1 in promoting osteoblast activity and inhibiting osteoclast activity [53]. In an animal model of obesity, vitamin MK-4 appeared to promote osteogenesis by influencing the levels of activated OC and osteoprotegerin levels while reducing circulating RANKL levels [53]. It is known that RANKL stimulates osteoclasts and bone resorption by being a receptor activator for the nuclear factor kappa-B ligand, whereas osteoprotegerin can counteract this effect. The inhibition of RANKL-induced osteoclastogenesis by MK-4 and MK-7 appears to be dose-dependent [54]. Interestingly, the anti-osteoporotic drug denosumab (Prolia) acts by a similar mechanism [55]. It has also been observed that MK-7 can reduce PTH-induced bone resorption [56].

Another mechanism explaining the effects of vitamin K2 on bone health appears to depend on its binding to the steroid and xenobiotic sensing nuclear receptor, SXR [57,58]. However, MK-4 was the only vitamin K homologue that was bound to SXR, thereby activating SXR [59]. In osteoblasts, MK-4 has been shown to activate the classical SXR target CYP3A4, in addition to MK-4’s impact on several genes and enzymes involved in bone formation, inter alia bone-specific alkaline phosphatase (ALP), osteopontin (OPN), and osteoprotegerin [57]. The SXR receptor appears to play an important role in bone maintenance as knockout mice for PXR, which corresponds to SXR in humans [58], showed enhanced bone resorption and developed severe osteopenia despite adequate dietary vitamin K [59]. Taken together, the existing clinical and laboratory data strongly indicate that appropriate vitamin K supplementation improves BMD and reduces fracture risk, especially in postmenopausal women. 

## 5. Mechanisms of Action of Vitamin D

Today, vitamin D3 has become an established agent in the prevention of osteoporosis. Recent meta-analyses have confirmed previous indications that supplementation with vitamin D3 significantly increases BMD and reduces fracture risk in people above 65 years old when administered at a dose of at least 20 µg/day [60,61,62]. 

The influence of vitamin D on bone functioning is not limited to the influence on Ca uptake and metabolism. It also includes impacts on osteoblast and osteoclast functioning. These effects are mediated by the nuclear vitamin D receptor [63,64,65]. Vitamin D exerts effects directly on osteoblasts by promoting osteoblast maturation and OC synthesis [66,67]. The multiple effects of vitamin D on bone are associated with a high expression of the vitamin D receptor in several types of bone cells [68]. 

Most of the actions of vitamin D are mediated by 1α,25-dihydroxyvitamin D, its activated form, through a nuclear transcription factor known as the vitamin D receptor [69]. Upon entering the nucleus of a cell, 1α,25-dihydroxyvitamin D binds to this receptor and recruits another nuclear receptor known as retinoid X receptor (RXR). The activated receptor complex then binds small sequences of DNA known as vitamin D responsive elements, thereby modulating the transcription of several genes. Activation of vitamin D takes place in the following two steps: 25-hydroxylation in the liver followed by 1-hydroxylation in the kidneys. However, local activation of 25-(OH)D_3_ to 1,25(OH)_2_D_3_ may also take place in osteoblasts, thereby exerting autocrine activity and the maintenance of bone tissue [70].

## 6. Clinical Assessment of Pre-Interventional Vitamin Status

Before beginning supplementation with vitamin D3 and/or vitamin K, it is recommended to conduct a clinical assessment of the vitamin status; however, several researchers and clinicians have recommended and used the vitamin supplementation in middle-aged and elderly individuals without an initial assessment of their vitamin status. Suboptimal vitamin levels are frequently seen in elderly segments of the population and may be accompanied by increased bone turnover and decreasing BMD. Circulating levels of 25(OH)-D3 are routinely used as the biomarker of vitamin D status. The U.S. Endocrine Society has suggested judging the vitamin D status as deficient or insufficient if serum values are below a limit of 75 nmol/L, and sufficient when serum 25-hydroxyvitamin D values are at or above this value. The U.S. Institute of Medicine (IOM) and other official institutions have recommended 50 nmol/L as the minimal level of sufficiency [71]. As for the estimation of vitamin K functional status, a low circulating level of carboxylated osteocalcin (cOC) relative to undercarboxylated osteocalcin (ucOC) or a low ucOC serum concentration seems to provide the most relevant information on insufficient vitamin K status. However, circulating levels of ucOC have been regularly found to be increased in postmenopausal as compared to pre-menopausal women, as well as to be markedly higher in women over the age of 70, indicating a decreased vitamin K activity in bone tissue [64]. A high ucOC-to-cOC ratio appears to be predictive of hip fracture risk in elderly women [29,43,48]. However, in routine clinical chemistry laboratory tests, only impaired blood coagulation is used as an imprecise and rough confirmative test on clinical vitamin K deficiency. Food frequency questionnaires as well as specific determinations of K1 and K2 levels in specialized laboratories can help to provide additional information.

## 7. Vitamin D and Vitamin K Cooperators in Bone Protection?

Supplementation with vitamin K2 on top of vitamin D3 supplementation has appeared to improve BMD in postmenopausal women [41]. Adequate intake or supplementation with vitamin D and vitamin K combined are reported to be key protective agents in the prevention of osteoporosis today. Thus, a case-control study that included 111 hip fracture patients and 73 controls (median age, 83 years) found that lower serum concentration of both 25-hydroxyvitamin D and vitamin K1 in patients compared to controls was associated with increased risk of hip fracture [72]. 

A meta-analysis from 2020 of eight randomized clinical trials enrolling 971 study participants concluded that vitamin K combined with vitamin D3 significantly increased the total bone mineral density [73]. Rønn et al. [74] conducted a placebo-controlled RCT using both MK-7 (375 μg/day) and vitamin D3 plus calcium for three years in 142 postmenopausal women with osteopenia. They found that the combination increased carboxylation of osteocalcin, when compared with the placebo group which only received vitamin D3 plus calcium. However, in this study, the changes in bone turnover biomarkers were similar between the sub-groups with and without supplemented vitamin K. 

In a recent RCT including 108 osteoporotic post-menopausal women with suboptimal vitamin K status, Moore and colleagues [75] investigated the effect of giving K1 (1 mg/day) or MK-4 (45 mg/day) in addition to bisphosphonate and calcium and vitamin D treatment (the latter combination referred to as placebo). In this study, there were no additional effects of vitamin K supplementation on BMD or bone turnover markers, but there was a modest effect of K1 on hip geometry.

In another RCT, 122 postmenopausal women were randomized into four groups [76]. Three groups were fed farmed salmon containing either high levels of vitamin D and K1 or high vitamin D and low K1 or low vitamin D and high K1, together with a calcium supplement. The fourth group was fed vitamin D and calcium. In all the groups, there was a positive effect on bone markers, but there were no significant differences between these groups. 

A 2017 literature review of animal and human studies suggested that the optimal concentrations of both vitamin D3 and vitamin K are beneficial for bone health in postmenopausal women [77]. The somewhat limited evidence (see also studies included in the meta-analyses of Ma et al., 2022 [41] and Huang et al., 2015 [45]) supports the hypothesis that combined vitamin D and vitamin K supplementation may be more effective than the supplementation with either vitamin alone for bone health. In animal models of osteoporosis, vitamin K has demonstrated osteoprotective effects. Specifically, in a rat study, vitamin K supplementation was shown to be more efficient in improving bone characteristics in a model of immobilization osteoporosis as compared to combined Ca and vitamin D supplementation [78]. A protective effect of MK-4 was also observed in a model of glucocorticoid-induced bone loss [79]. 

## 8. Dietary Sources and Pharmacokinetics of the Vitamins K and D

The sources of vitamin K are different depending upon the vitamer. Vitamin K1 is found mainly in dark green leafy vegetables, such as kale, spinach, and broccoli, where it is bound to the membranes of the chloroplasts [80]. Menadione (K3) is often added as a supplement in animal feed and is converted to MK-4 in the animals’ liver and peripheral tissues, which gives MK-4 in animal food products. Fermented foods including fermented butter or cheese are sources of vitamin K2, especially the long-chain variants in the series MK5–10, depending on the starter culture in the cheese [25]. 

Bioavailability of K1 depends on the food’s matrix. Thus, uptake of the vitamin from meals of pure spinach or broccoli is only 5–10%, but the uptake is doubled with fat in the same meal [25,80]. It has been estimated that about 80% of the intake of vitamin K is as K1 worldwide [80,81]. Natto, a traditional Japanese soybean-based food, produced by fermentation, is an important source of MK-7 [81]. 

It is known that the vitamins K and D from food and supplements are incorporated into mixed micelles of lipids in the small intestines via the action of bile and pancreatic enzymes. The vitamins are absorbed together with the lipoid compounds in the small intestine [81,82]. 

Both the K1 and the K2 are absorbed via the enterocytes with the help of bile salts. From the blood circulation, vitamin K is taken up by the liver, where it is metabolized. Patients who have an ileostomy or those who have undergone bariatric surgery are at risk of deficiency of the vitamin [6]. But these patients can still absorb supplemental vitamin K2 administered orally, provided a sufficient dose is given [83,84]. 

After the intake of vitamin K2, Møller et al. [44] found a maximal blood serum concentration after five hours. However, the concentration did not return to the pre-administration level within the 72 h observation period [83], presumably due to the reaction of vitamin K in the carboxylation of vitamin K-dependent proteins and inefficient recycling of epoxidized vitamin K. In 2017, the EFSA NDA panel concluded that dietary vitamin K2 MK-7 is more efficiently absorbed than synthetic free vitamin K1 [84]. However, it should be noted that dietary vitamin MK-7 does not contribute much to dietary vitamin K intake in most of the European countries. Thus, a significant difference for serum MK-7 was seen in women from Tokyo (5.3 ng/mL), Hiroshima (1.2 ng/mL), and Britain (0.37 ng/mL), with a corresponding inverse correlation with incidence of hip fractures, where the intake of natto was the only correlated food eaten that contributed MK-7 [85]. 

**Figure 3:**
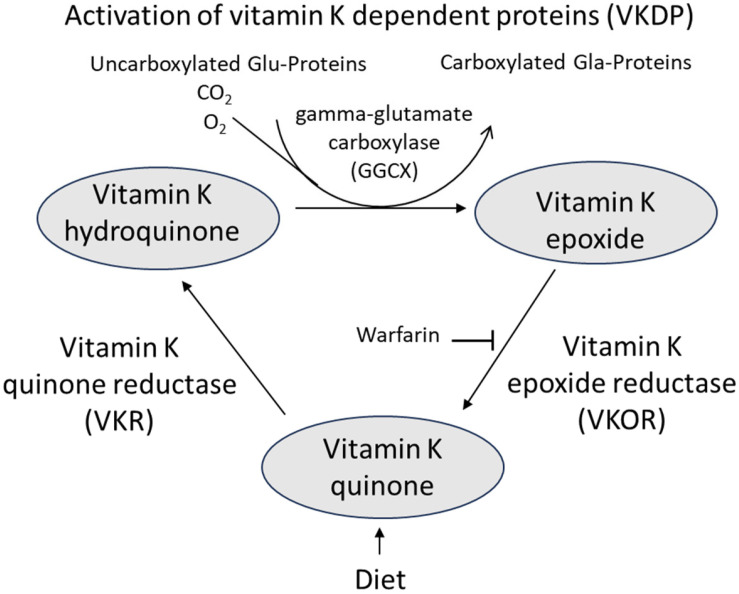
The vitamin K cycle resulting in enzymatic reactivation of oxidized vitamin K. The cycle consumes reducing equivalents in the form of NAD(P)H. In the presence of warfarin, reactivation of vitamin K is inhibited, and instead vitamin K epoxide is degraded [89] Liu et al., 2023.

It should be noted that the drug ezetimibe that is used for reducing cholesterol uptake from the gut also inhibits the vitamin K uptake [86]. High-dosed statin therapy may also be a risk factor for osteoporosis [87] since statin therapy leads to the inhibition of the enzyme, HMG CoA reductase, that is necessary for the synthesis of the MK-4 vitamer, which is prevalent in peripheral tissues [22,88]. It should also be noted that the anticoagulant drug, warfarin, that inhibits vitamin K epoxide reductase in the vitamin K cycle (Figure 3) may lead to vitamin K deficiency because of reduced recycling, also in bone tissue, and may thereby promote the development of osteoporosis [89]. More recently, warfarin has been gradually replaced by direct acting oral anticoagulants without this general impairing effect on all VKDP [89].

As discussed, a functional indicator for assessing the vitamin K status is the ratio of cOC-to-ucOC or ucOC concentration. It has been reported that a supplemental intake of 180–250 μg/day of vitamin K1 activated osteocalcin appropriately [90]. There is no conclusive information on the mechanism of uptake of vitamin K by the bones. However, it is known that the vitamin can be reactivated enzymatically by the vitamin K cycle [89] and reused after its oxidation during carboxylation of osteocalcin and other proteins (Figure 3). 

During pregnancy, only small quantities of vitamin K1 cross the placenta from mother to fetus. Blood concentrations of K1 in the full-term newborn are about half of that of the mothers. The K1 concentration in cord blood is as low as <0.1 nmol/L [81], explaining the importance of parenteral vitamin K supplementation to the newborn. 

In 2017, the EFSA NDA panel [84] estimated that a mean of about 60% of injected K1 is excreted in urine and feces. No similar experiment has been carried out to assess the losses of metabolites after vitamin K2 ingestion.

With respect to vitamin D, an inactive precursor is cholecalciferol that must be converted to biologically active forms in the liver and kidneys. Following dietary intake or synthesis in the skin, the vitamin D precursors enter the circulation and are transported to the liver by the vitamin D-binding protein. In the liver, these precursors are converted to 25-hydroxyvitamin D, which makes up the major circulating form of the vitamin [91]. In the kidneys, the circulating compounds are hydroxylated to 1,25(OH)_2_D_3_ (1α,25-dihydroxyvitamin D/calcitriol). The production of this form in the kidneys is regulated by several factors, including serum calcium and parathyroid hormone (PTH). Most of the physiological effects of vitamin D in the body are related to the activity of calcitriol. Vitamin D acts to maintain Ca homeostasis in plasma. Therefore, the action of vitamin D on bone is complicated, as it may both stimulate mineralization and, in situations of low blood Ca, also promote osteoclasts to mobilize Ca from bone tissue [91]. 

## 9. Conclusions and Perspectives

There exists an extensive body of evidence related to the positive effect of adequate vitamin K status and of vitamin K2 supplementation regarding the carboxylation of osteocalcin and bone health. Little is known about the efficacy of vitamin K1 supplementation, as most supplementation studies have been conducted with vitamin K2. Many studies have also used high and pharmacological doses of vitamin K2. Further research is necessary on the various mechanisms of actions of these vitamers for establishing vitamin K2 as a safe and cost-effective supplement for prevention of osteoporosis. In particular, there is a need for more research into questions related to the synergistic role of calcium and the vitamins D3 and K with respect to bone health. At present, it might be anticipated that the vitamins K and D3 would provide a preventive role, particularly in aged people. A combined regimen including vitamin K might also be of benefit in the prevention of osteoporosis following bariatric surgery as vitamin D3 and Ca do not seem to suffice. As for the treatment of the manifestation of osteoporosis, drugs such as bisphosphonates or denosumab will still be essential as vitamin K2 appears to have limited additional effect on BMD.

However, there remains the need for research to establish data on optimized intakes and the ratio of vitamin K to vitamin D3, as well as data for optimal blood levels of the two protectors when supplemented in combination to individuals at risk. As for the determination of the vitamin K status, the most reliable measure may be the ratio of total to undercarboxylated osteocalcin levels. It should be taken into consideration that the optimal vitamin K supplementation may vary from individual to individual.

Finally, there is a need for additional research into the long-term effects of combined supplementation with vitamin K and vitamin D3. Since the burden of osteoporosis is about to increase worldwide, this research into preventive measures should be given high priority. 

## Data Availability

The data used in this article are sourced from materials mentioned in the References section.
